# Generation and characterization of a new mammalian cell line continuously expressing virus-like particles of Japanese encephalitis virus for a subunit vaccine candidate

**DOI:** 10.1186/1472-6750-14-62

**Published:** 2014-07-10

**Authors:** Rong-Hong Hua, Ye-Nan Li, Zhen-Shi Chen, Li-Ke Liu, Hong Huo, Xiao-Lei Wang, Li-Ping Guo, Nan Shen, Jing-Fei Wang, Zhi-Gao Bu

**Affiliations:** 1 State Key Laboratory of Veterinary Biotechnology, Harbin Veterinary Research Institute, Chinese Academy of Agricultural Sciences, Maduan Street, Harbin 150001, PR China

**Keywords:** Japanese encephalitis virus, Mammalian cell line, Virus-like particle, Subunit vaccine

## Abstract

**Background:**

Japanese encephalitis virus (JEV) is the most important cause of epidemic encephalitis in most Asian regions. There is no specific treatment available for Japanese encephalitis, and vaccination is the only effective way to prevent JEV infection in humans and domestic animals. The purpose of this study is to establish a new mammalian cell line stably and efficiently expressing virus-like particle of JEV for potential use of JEV subunit vaccine.

**Results:**

We generated a new cell clone (BJ-ME cells) that stably produces a secreted form of Japanese encephalitis virus (JEV) virus-like particle (VLP). The BJ-ME cells were engineered by transfecting BHK-21 cells with a code-optimized cDNA encoding JEV prM and E protein expression plasmid. Cell line BJ-ME can stably produces a secreted form of Japanese encephalitis virus virus-like particle (JEV-VLP) which contains the JEV envelope glycoprotein (E) and membrane protein (M). The amount of JEV-VLP antigen released into the culture fluid of BJ-ME cells was as high as 15–20 μg/ml. JEV-VLP production was stable after multiple cell passages and 100% cell expression was maintained without detectable cell fusion or apoptosis. Cell culture fluid containing the JEV-VLP antigen could be harvested five to seven times continuously at intervals of 4–6 days while maintaining the culture. Mice immunized with the JEV-VLP antigen with or without adjuvant developed high titers of neutralizing antibodies and 100% protection against lethal JEV challenge.

**Conclusion:**

These results suggest that the recombinant JEV-VLP antigen produced by the BJ-ME cell line is an effective, safe and affordable subunit Japanese encephalitis vaccine candidate, especially for domestic animals such as pig and horse.

## Background

Japanese encephalitis virus (JEV) is the most important cause of epidemic encephalitis in most Asian regions, with about 35,000–50,000 cases of and 10,000 deaths from JEV infection reported annually [1]. The virus can be found in regions beyond its ecological boundaries, with recent reports of JEV having spread as far as northern Australia [2-4] and Pakistan [5]. Hence, there is concern that JEV might become a global threat.

Japanese encephalitis (JE) caused by JEV is a mosquito-borne zoonotic infectious disease [6,7]. A variety of animals are susceptible to JEV infection, but usually only humans, horses and pigs with infection exhibit symptoms [8-11]. Humans and horses are generally thought to be dead-end JEV hosts [12,13]. Pigs are considered the most important amplification and reservoir hosts of JEV in endemic regions [7,13]. JEV infection in pregnant sows can cause abortion, stillbirth and other reproductive failure. In addition, infection of boars can cause acute testicular inflammation and hypospermia [14,15].

There is no specific treatment available for JE, and vaccination is the only effective way to prevent JEV infection in humans and domestic animals. At present, there are three kinds of JE vaccines available for humans: the live attenuated virus vaccine SA14-14-2 [16-19], Vero cell-derived formalin-inactivated whole-virus SA14-14-2 strain vaccine IC51 and Beijing-1 strain vaccine JEBIKV [19-21], and recombinant chimeric virus vaccine JE-CV [22,23]. These vaccines have better safety profiles and fewer side effects compared with the mouse brain-derived inactivated vaccine [24-26]. The attenuated live vaccine is derived from primary hamster kidney cells, is difficult and costly to manufacture, and the infectious agents are always associated with potential biosafety issues.

The use of infectious agents is always a major issue with manufacturing process of the currently used vaccines. Therefore, the development of new types of JE vaccines for humans and domestic animals that do not involve the use of infectious JEV is imperative. Genetically engineered vaccine is a potential new form of the JE vaccine. Previous studies have shown that the pre-membrane (prM) and E protein expressed in mammalian cells could assemble into virus-like particles (VLP), and that the expressed JEV VLP has the same ability to induce neutralizing antibodies and protective efficiency as the JEV virion [27-30]. Thus, studies on second-generation JE vaccines have focused on the production of the JEV-VLP antigen by genetic engineering of a stable cell line. Different mammalian cell lines including RK13 [31], COS-1 [32] and CHO-k1 [33] have been used to construct stable cell lines that continuously express JEV-VLP, but do not express efficiently or have additional defects.

In this study, we have generated a new cell line, BJ-ME, which can stably produce a secreted form of the JEV VLP by using BHK-21 cells as the parent cell line. Mice immunized with the expressed JEV-VLP antigen developed high titres of neutralizing antibodies and complete protection against lethal JEV challenges.

## Results

### Establishment of a stable cell clone continuously expressing the prM/M-E antigen

**Figure 1:**
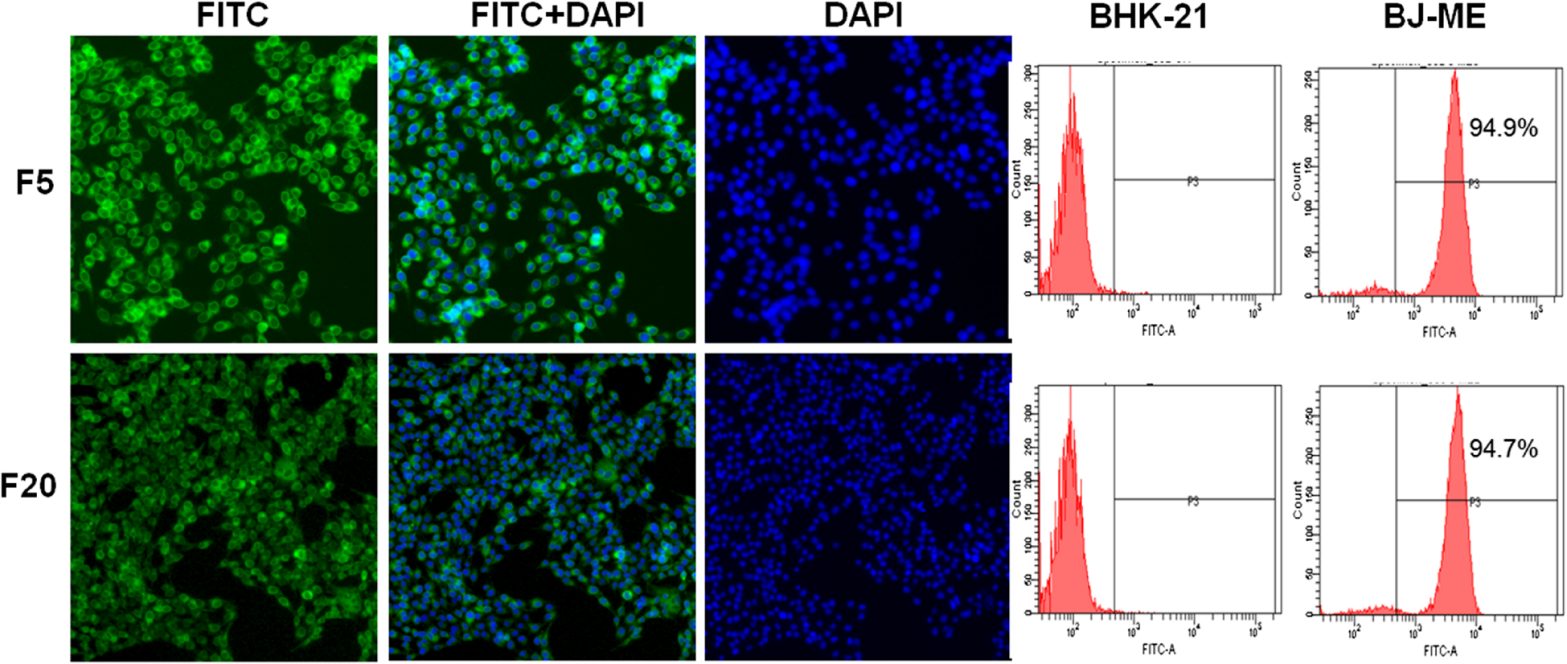
**Immunofluorescence and flow cytometry analysis of BJ-ME in the 5th and 20th generation with monoclonal antibody against the JEV E protein.** The percentages of cells expressing E protein in the 5th and 20th generation of BJ-ME cells were all over 94%.

**Figure 2:**
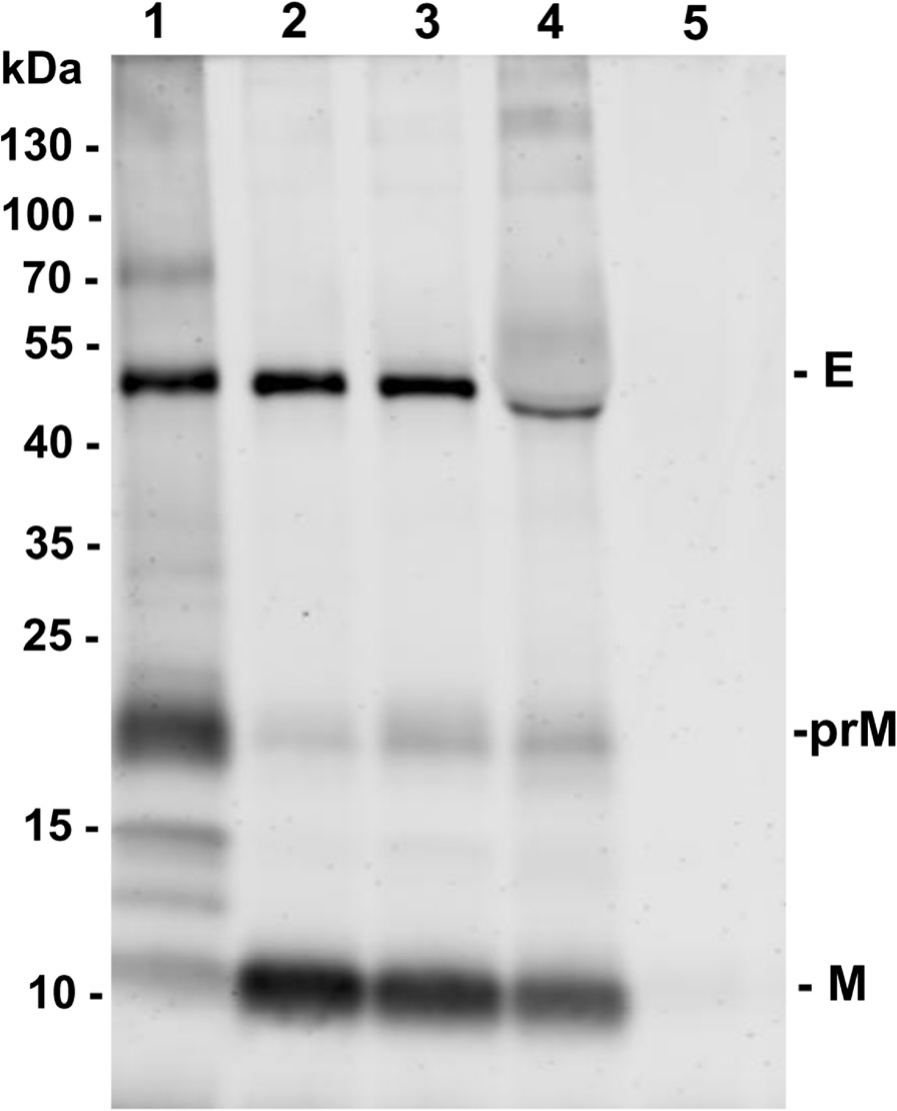
**Cell lysates and culture supernatants were analyzed by Western blotting with MAbs (5E7 and 3C8).** 1, Cell lysates of BJ-ME cells; 2 and 3, Culture supernatants of BJ-ME cells. 4, JEV control. 5, Cell lysates of BHK-21 cells. Relative locations of E, prM and M protein of JEV were indicated on the right.

**Figure 3:**
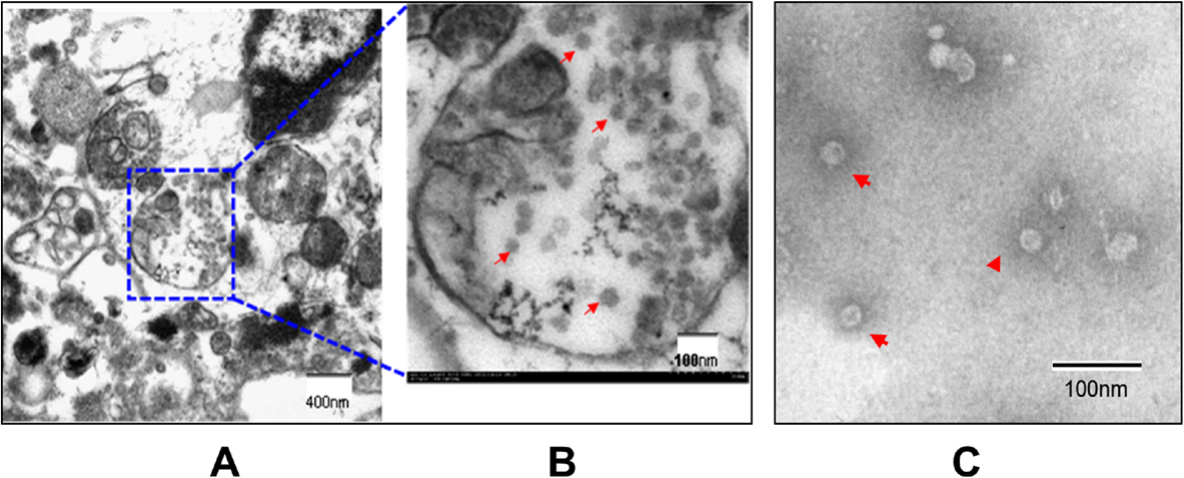
**Electron micrographs of BJ-ME expression of JEV VLP. (A)** Low-magnification image of BJ-ME cells. **(B)** Higher magnification image of BJ-ME cells. Virus-like particles in the ER of BJ-ME cells are indicated with arrowheads. **(C)** JEV VLP purified from the culture supernatant of BJ-ME cells. The purified antigen was processed for negative staining and observed with an electron microscope.

From the cloned colonies of BHK-21 cells, 64 G418-resistant and E protein-specific IFA-positive cell colonies were picked and transferred to 24-well plates. After comparison of E protein expression in the culture medium of the 64 cell colonies by Western blotting, 10 clones with relatively high E protein expression were selected for further comparison. After IFA testing and further cloning purification, the E protein expression efficiency of all 10 cell clones was compared using E protein-specific Western blotting. The clone of most efficiently expression E antigen was selected and designated as BJ-ME (BHK-JEV-ME). The cells were expanded and maintained in G418-containing medium to observe JEV antigen production. The BJ-ME cell clone had undistinguishable morphology from parental BHK-21 cells and induced no polykaryocyte formation. When the fifth-passage BJ-ME cells were examined by indirect immunofluorescence and flow cytometry, over 94% of BJ-ME cells were positively stained with JEV E protein-specific MAb 5E7. This high percentage of E-expressing cells was maintained till the twentieth passage (Figure 1).Western blotting results with the MAbs 5E7 and 3C8 showed that BJ-ME cells expressed the prM and E protein together, and the majority of prM in the cells did not show cleavage. When secreted into culture medium, most of the prM protein was cleaved by furin into mature M protein (Figure 2).The morphology of BJ-ME cells was further examined by transmission electron microscopy. The results showed that VLP structure was observed in the endoplasmic reticulum of cells (Figure 3A,B), and this JEV-VLP structure was further confirmed with purified JEV-VLP by negative staining electron microscopy observation (Figure 3C). The appearance of the JEV-VLP was in the form of particles with an average diameter of 40–50 nm surrounded by a lipid bilayer.

### Passage stability and antigen expression of BJ-ME cells

For BJ-ME cells, passage 1 was defined as the cell culture from the 96-well plate by limiting dilution cloning and with 100% E expression. After expansion and cryopreservation, the culture medium of BJ-ME cells cultured in 75 cm^2^ flasks was collected from passage 5. The JEV-VLP was purified by ultrafiltration and rate zonal centrifugation. The purified antigen was used in negative staining electron microscopy observation and used as the antigen standard in an antigen capture ELISA quantification assay.

**Figure 4:**
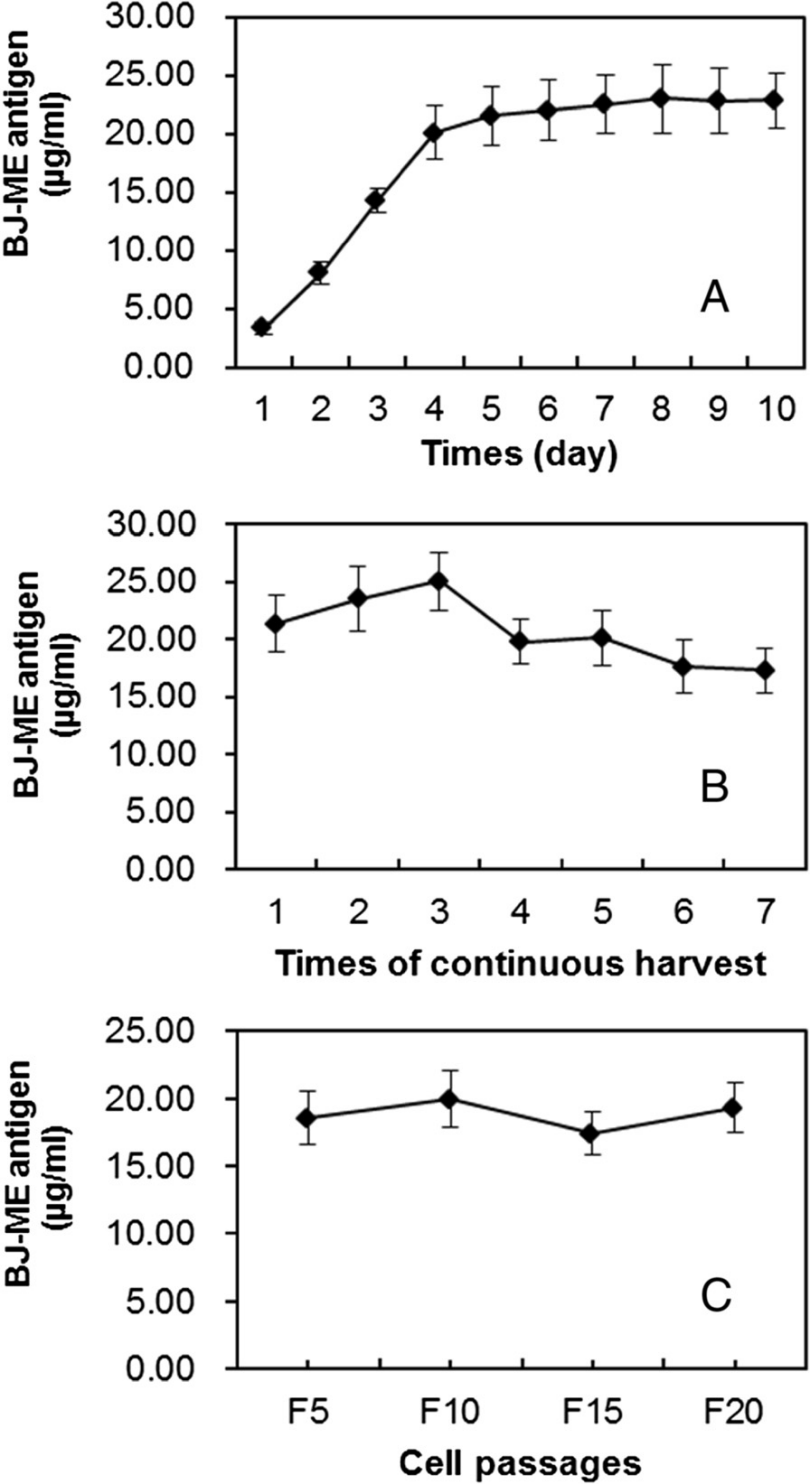
**Production of the JEV-VLP antigen by BJ-ME cells. (A)** Confluent BJ-ME cells were incubated with the maintaining medium for 10 days. The amount of JEV-VLP antigen produced each day (24 h) was determined by ELISA. **(B)** The culture medium of confluent BJ-ME cells was refreshed every 4 to 6 days continuously, and the amount of JEV-VLP antigen in culture supernatants harvested multiple times was determined by ELISA. **(C)** Effect of long-term passage on production of JEV-VLP antigen by BJ-ME cells. BJ-ME cells were passaged every 3 days in a 75 cm^2^ flask, and the amount of antigen produced by BJ-ME cells from the different passages was analyzed using ELISA.

To evaluate the antigen expression dynamics of BJ-ME cells, the confluent BJ-ME cells were passaged 1:5 into 75 cm^2^ flasks with 20 ml medium containing 10% FBS. For antigen amounts detection, in next day, the cells started growing in confluence and the medium containing 10% FBS were changed into maintaining medium (containing 1% FBS). Twenty four hours later, 600 μl of the culture medium was replaced with fresh serum-free medium every day. The amount of JEV-VLP was determined by ELISA. As shown in Figure 4A, the amount of JEV-VLP in the culture medium kept increasing in the first four days. From the fifth day, it reached a plateau, after which it started showing a slight increase from the fifth to tenth day. Cell culture fluid containing the JEV-VLP antigen could be harvested five to seven times continuously at intervals of 4 to 6 days during culture, and the concentration of JEV-VLP antigen collected at each harvest was over 15 μg/ml (Figure 4B).

The stability of JEV-VLP antigen expression by BJ-ME cells was further evaluated using cryopreserved cells. After thawing, BJ-ME cells were passaged every 2 days in 75 cm^2^ flasks. For antigen testing, the culture medium of confluent cells were changed into fresh maintaining medium. 4 to 5 days later the culture medium was collected at the 5^th^, 10^th^, 15^th^, and 20^th^ passages and examined for the amount of JEV-VLP antigen by ELISA (Figure 4C). Although the amount of JEV-VLP antigen produced by BJ-ME cells differed little between passages, more than 15 μg/ml of the JEV-VLP antigen was detectable throughout the monitoring period. Even at the 20^th^ passage, the frequency of E-expressing cells was over 94% (Figure 1).

### Immunogenicity and protective efficacy of the JEV-VLP

**Figure 5:**
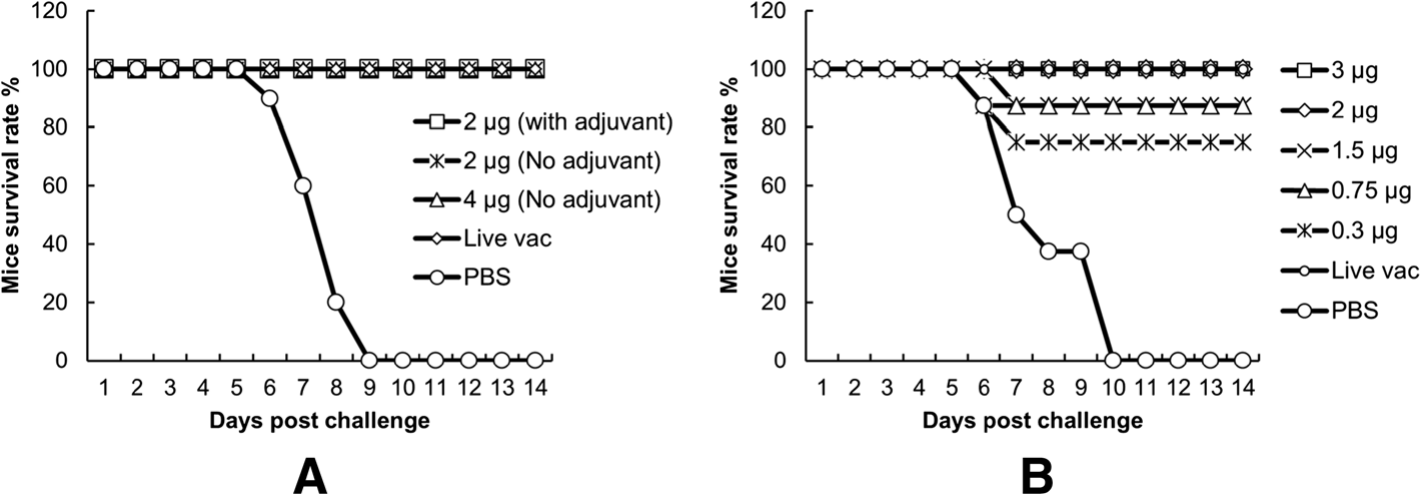
**Protection of mice against challenge with JEV. (A)** Groups of 10 4-week-old BALb/c mice were vaccinated as in Table 1 experiment 1. Immunized mice in all groups of JEV-VLP with or without adjuvant survived in 100%. **(B)** Survival rate of different dosage of JEV-VLP immunized mice. All mice were challenged with 1.0 × 10^7^ PFU of the P3 strain of JEV.

Mice administered the JEV-VLP with or without adjuvant developed high titers of neutralizing antibody. Even the neutralizing antibody titer in mice that were not administered the adjuvant was somewhat higher than that observed in mice immunized with the live virus vaccine (Table 1). All mice immunized with the JEV-VLP with or without adjuvant and the live virus vaccine were completely protected against JEV. All mice that were administered PBS died within 10 days after the challenge (Figure 5A).

**Table 1:** Induction of neutralizing antibodies in mice vaccinated with the JEV-VLP antigen

**Expt**	**Vaccine**	**Dosage**	**Neutralizing antibody titer**^**a**^		**Survival rate** ** (no. alive/total)**
			**Day 14**	**Day 28**	
1	BJ-ME^b^ (oil adjuvant)	2 μg	40	100	100% (10/10)
	BJ-ME (no adjuvant)	2 μg	70	100	100% (10/10)
	BJ-ME (no adjuvant)	4 μg	100	280	100% (10/10)
	Live vac^c^	10^6^ TCID_50_	100	120	100% (10/10)
	PBS	200 μL	<10	<10	0% (0/10)
2	BJ-ME 200 μl	3 μg	ND	160	100% (8/8)
	BJ-ME 150 μl	2.25 μg	ND	140	100% (8/8)
	BJ-ME 100 μl	1.5 μg	ND	100	87.5% (7/8)
	BJ-ME 50 μl	0.75 μg	ND	70	87.5% (7/8)
	BJ-ME 20 μl	0.3 μg	ND	40	75% (6/8)
	Live vac	10^6^ TCID_50_	ND	160	100% (8/8)
	PBS	200 μL	ND	<10	0% (0/8)

^a^Titers are expressed as the maximum serum dilution yielding a 50% plaque reduction. ^b^JEV-VLP antigen. ^c^Live virus vaccine (SA14-14-2 strain). ND, not determined.

For the second experiment, the immunogenicity and protective efficacy of different doses of the antigen were evaluated. As shown in Table 1, mice in the groups administered 3 μg and 2 μg of the antigen developed high levels of neutralizing antibody similar to that of the live vaccine group and were completely protected against the JEV challenge. Mice in the groups administered 0.3–1.5 μg of the antigen developed relatively lower titers of neutralizing antibody, and were partially protected against JEV; however, the survival rate in all groups was higher than 70% (Table 1, Figure 5B).

## Discussion

There is an urgent need for an effective, affordable and safer JE vaccine for humans and domestic animals. Biotechnically-engineered cell lines expressing the JEV-VLP antigen may meet this requirement. Here, we describe another alternative, a recombinant subunit vaccine candidate, based on a new mammalian cell line.

The BJ-ME cells described in this study produce extremely high levels of the JEV-VLP antigen—15–20 μg/ml—which is higher than that of any other reported cell lines such as J12#26 [31], F [33] and JE-4B [32]. This value was determined under experimental conditions involving simple changes in the culture medium, and we believe that the titer could be further increased under optimal culture conditions. Moreover, time-course results suggest that 5 days of incubation is sufficient for antigen production.

BJ-ME cells had similar cell morphology and growth characteristics as the parental BHK-21 cells. In fact, cell clones with different morphogenetic characteristics found during the selection and cloning process were found to release the E antigen inefficiently. This phenomenon indicates that maintaining similar morphology and growth characteristics with the parental cells may be important in the engineering of cell lines with high efficiency and stable exogenous gene expression. Another factor that may have contributed to the high efficiency of JEV-VLP antigen expression in BJ-ME cells is the genetic codon optimization of the cDNA sequence encoding the prM and E proteins. Different species have codon usage bias, and optimizing the genetic codon according to the bias of the expressing host could increase the expression efficiency of the target gene [34-36]. The BJ-ME cells showed stable JEV-VLP production during cell culture and passage. Even after 20–25 passages, the JEV-VLP antigen-producing efficiency of BJ-ME cells was not significantly reduced.

A major obstacle to the stable expression of flavivirus membrane protein in mammalian cells is its cytotoxicity. In a previous report, attempts to establish cells with high expression of the prM-E protein from Vero cells were unsuccessful [31]. This is probably because Vero cells are susceptible to JEV infection, which makes them sensitive to the cytotoxic effects of the JEV-VLP antigen. Although BHK-21 cells are susceptible to JEV infection, we did not observe any obvious cytotoxic effects of JEV VLP. It was possible to obtain dozens of G418-resistant cell colonies with a single transfection and selection cycle. The cell line BJ-ME constructed in this study has the same growth characteristics as the parent cell line BHK-21. So this cell line could easily be cultured in serum-free or low-serum medium. J12#26 cells derived from RK13, a rabbit kidney cell line, was the most stable and highest antigen-producing cell line in all previous reports [31]. However, RK13 cells grow slowly and could not reach high cell density, so are not suitable for large-scale culture. Another important point is that RK13 cells contain bovine viral diarrhea virus [37], which make them unsuitable for vaccine antigen production.

To overcome the fusogenic cytotoxicity of JEV prM-E protein expression in mammalian cell lines, Konishi et al. [33] introduced a mutation at the prM/M cleavage site to prevent authentic processing and to reduce the toxic fusion activity of the expressed VLP, and established an F-cell line using CHO-k1 as the parent cell line. The elimination of cleavage of the prM protein resulted in the production of immature VLPs, which are quite different in their conformational structure from the mature VLPs produced by authentic cleavage of prM [38]. These structural differences may eliminate virus conformational neutralizing epitopes. Moreover, reports have shown that most JEV-neutralizing epitopes are conformation dependent [39]. The differences in the conformational structure will affect the immunogenicity of the F antigen. In fact, the protection rate in mice immunized with the F antigen with uncleaved prM protein did not exceed 50% [33]. Recently Yamaji et al. further reported expressing the JEV prM protein mutated virus-like particles with baculovirus [40] and lepidopteran insect cell lines [41]. Obviously the yield of E antigen were increased in both of two expressing systems, especially the E antigen yield reached about 30 μg/ml in lepidopteran insect cell line. However, these studies did not present immune protection result of these immature JEV virus-like particles with uncleaved prM protein. The differences in structural and immune protective effect between immature and mature JEV virus-like particles need further evaluation. Maybe DENV-related reports [42,43] could provide some clues for future survey the relation of immune protective effect and structure of virus-like particles.

As expected be of particulate form, the BJ-ME antigen was immunogenic. With or without adjuvant, dose of 2 μg of BJ-ME induced high titers of neutralizing antibodies and provided complete protection against challenge. However we observed no enhancement effect of the adjuvant in this experiment which may because the antigen amount of 2 μg is overdosed for mice. Another experiment with different dose of antigen showed that 0.3 μg of BJ-ME antigen could induce effective protection neutralizing antibodies titer of 1:40 which is higher than surrogate maker for seroprotection of neutralizing antibody titer of 1:10.

## Conclusions

The new established cell line BJ-ME could efficiently and stably produces secreted form of JEV-VLP. BJ-ME cells expressing the JEV-VLP antigen showed good immunogenicity in mice. An antigen dose of at least 0.3 μg could induce neutralizing antibody production, and more than 70% of immunized mice survived after lethal challenge with JEV. These results suggest that the recombinant JEV virus-like particle antigen produced by the BJ-ME cell clone is an effective, easy to produce, and safe second-generation subunit JE veterinary vaccine candidate. Moreover, the JEV-VLP could be used to make diagnostic reagents for JE and JEV.

## Methods

### Animal ethics

Care of laboratory animals and animal experimentation were performed in accordance with animal ethics guidelines and approved protocols. All animal experiments were approved by the Animal Ethics Committee of Harbin Veterinary Research Institute of the Chinese Academy of Agricultural Sciences. The Animal Ethics Committee approval number was SYXK (Hei) 2011–022.

### Cells and viruses

Baby hamster kidney BHK-21 cells (CCL-10; ATCC) were cultured in Dulbecco’s modified Eagle’s medium (DMEM; Gibco, Invitrogen, Carlsbad, CA) supplemented with 10% fetal bovine serum (FBS; Gibco, Grand Island, NY). C6/36 cells (CRL-1660; ATCC) were cultured at 28°C in a 5% CO_2_ atmosphere in RPMI-1640 (Gibco, Grand Island, NY) supplemented with 10% FBS. The attenuated JEV vaccine strain SA14-14-2 was propagated and titrated on BHK-21 cell monolayers, and stored at -80°C until use. The P3 strain of JEV used to challenge mice was propagated in mouse brains. The P3 strain of JEV used in the plaque reduction neutralizing assay was passaged through C6/36 cells twice, and then passaged twice through BHK-21 cells.

### Construction of plasmids carrying the prM and E genes of JEV

First, a genetic codon-optimized JEV cDNA encoding the viral signal peptide of the carboxyl terminus of the C, prM, and E protein of the SA14-14-2 strain (amino acid positions 106 to 794, GenBank: **AAK11279.1**) was synthesized and cloned into the pUC57 plasmid. Then DNA fragment was subcloned into the the expression vector pCAGneo to generate pCAGneo-opti-JEV-ME. The plasmid pCAGneo contains the neomycin resistance gene, which confers resistance to G418. The resulting plasmid pCAGneo-opti-JEV-ME was used to transfect stable cell lines.

### Establishment of stable cell lines constitutively producing the E antigen

Monolayer or subconfluent BHK-21 cells were transfected with the pCAGneo-opti-JEV-ME plasmid using FuGENE HD transfection reagent (Roche Diagnostic GmbH, Mannheim, Germany). Two days later, the transfected cells were digested and cloned by limited dilution in 96-well plates and growth in medium containing G418 (1000 μg/ml). The cloned cells were selected by Indirect immunofluorescence assay (IFA) with JEV E protein-specific monoclonal antibody 5E7 (unpublished data). The amount of E antigen in culture supernatants from cloned cells was examined and compared by Western blotting. One clone designated BJ-ME (BHK-JEV-ME) that showed obviously more efficient expression of the E antigen was selected and maintained for further characterization and antigen production.

### Indirect immunofluorescence assays and flow cytometry analysis

The cells were washed with phosphate-buffered saline (PBS), fixed with 4% paraformaldehyde at room temperature for 20 min, washed and then premeabilized with PBS containing 0.1% Triton X-100 (PBS-T). Then the cells were probed with JEV E protein-specific MAb 5E7 or JEV prM/M protein-specific MAb 3C8 at the procedure described before [44]. And cell nuclei were stained with DAPI (4′,6-Diamidino-2-phenylindole dihydrochloride). Cells were then observed under a fluorescence microscope (IMT2 Olympus, Tokyo, Japan). For flow cytometry analysis, the cells were dispersed by trypsin digestion and then fixed as above described.

### Antigen purification

The culture medium collected at 4–6 days after subculture of BJ-ME cells was clarified by centrifugation. The clarified medium was concentrated 40-fold in volume by ultrafiltration with a membrane with a size exclusion limit of 100 kDa (Biomax100; Millipore, Bedford, MA, USA). The concentrate was subjected to rate zonal centrifugation in 10–50% (wt/wt) linear sucrose gradients. Fractions were collected and analyzed by SDS-PAGE and Western blotting for the E antigen. Fractions of 20–30% sucrose were collected and pooled. Sucrose was removed from the E antigen pool by Sephadex G-25 chromatography with the NAP-25 column (GE, Westborough, MA, USA), equilibrated and eluted with PBS. The purified JEV-VLP in PBS was quantified with quick-start Bradford protein assay kits (Bio-Rad Laboratories Inc., Hercules, CA, USA) according to the manufacturer’s instructions.

### Western blotting analysis

JEV-VLP antigen in BJ-ME cell culture supernatants or cell lysates were analyzed by Western blot analysis at the procedure described previously [44]. The membrane was probed with MAb 5E7 or/and 3C8.

### Antigen capture ELISA for quantification of antigen

The amounts of the BJ-ME antigen were quantified by a sandwich ELISA with a pair of JEV specific MAb. 5E7 and 5B10 which were generated with purified BJ-ME antigen according to the procedure described previously [44-46]. Briefly MAb 5B10 (2 μg/ml) was coated on 96-well plates overnight at 4°C, and blocked with 5% skimmed dried milk for 2 h at 37°C. Subsequently, the plates were washed three times with phosphate-buffered saline with 0.1% Tween-20 (PBST). In the binding assay, the plates were incubated with the various dilutions of samples at 37°C for 2 h followed by washing three times with PBST. Bound antigens were detected with horseradish peroxidase (HRP)-conjugated MAb 5E7. After additional incubation 37°C for 1 h and thorough washing with PBST, the reactions were developed with TMB substrate. The reaction was stopped with 2 M H_2_SO_4_, and absorbance was measured at 450 nm using a microplate autoreader (Bio-Rad, Hercules, CA, USA). The results are presented as the average of duplicate assays. A stock of purified BJ-ME antigen was serially twofold diluted and used as standards. The antigen amounts of each sample were determined from the standard curve.

### Electron microscopic observation

Virus-like particles in BJ-ME cells were examined with transmission electron microscope as reported procedure [31]. For observation of BJ-ME cells expressing VLP in the culture supernate, the purified JEV-VLP was processed for negative staining on copper formvar-coated grids. Specimens were stained with sodium phosphotungstate and observed under an electron microscope.

### Immunization and challenge of mice

The supernates of BJ-ME cells cultured for 6 days without FBS were harvested and used for immunization. Supernates containing 20 μg/ml antigen emulsified with the same volume of MONTANIDE ISA50V2 (SEPPIC S.A., Paris, France) were used for immunization with a 200 μl dose to each mouse. Other two groups of 4-week-old female BALB/c mice were immunized subcutaneously with a 200 μl (for 4 μg antigen without adjuvant group) and 100 μl (for 2 μg antigen without adjuvant group) dose given to each mouse. PBS and 10^6.5^ TCID_50_/0.2 ml of the attenuated JEV vaccine strain SA14-14-2 given at the same dose were used as the negative and positive control, respectively. Two weeks after the first immunization, an enhanced immunization was given. Except for only once immunization was given for the live virus vaccine group. Mice were bled at intervals of 2 weeks for up to 4 weeks after the first immunization. Pooled serum samples from each group were stored for virus neutralizing antibody testing. Mice were challenged with intraperitoneal injections at the day after the last bleed with a 1.0 × 10^7^ PFU of the P3 strain of JEV and observed for 2 weeks.

In another experiment, the supernates containing different amount of antigen (0.3 μg to 3 μg per mouse) without adjuvant were used as immunogen. Groups of eight 4-week-old BALB/c mice were immunized, bled and challenged as described above. PBS and the live virus vaccine control groups were also conducted as described above.

### Neutralizing antibody testing

Virus neutralizing antibodies present in the mouse and pig sera were tested using a plaque reduction assay [31]. Except that BHK-21 cells were used rather than Vero cells. Neutralizing antibody titers were expressed as the reciprocal of the serum dilution yielding a 50% reduction in the mean plaque number versus that in the control wells.

## Competing interests

The authors declare that they have no competing interests.

## Authors’ contributions

RHH and ZGB designed the experiment. RHH, YNL, ZSC, LKL, HH, XLW, LPG, NS and JFW performed the experiments. RHH and ZGB analysed data and wrote the paper. All authors read and approved the final manuscript.
